# Development and validation of nomograms to predict survival of primary adrenal lymphoma: a population-based retrospective study

**DOI:** 10.1038/s41598-023-41839-2

**Published:** 2023-09-02

**Authors:** Shiwei Sun, Yue Wang, Wei Yao, Peng Yue, Fuyu Guo, Xiaoqian Deng, Jiandong Zhang, Yangang Zhang

**Affiliations:** 1 Third Hospital of Shanxi Medical University, Shanxi Bethune Hospital, Shanxi Academy of Medical Sciences, Tongji Shanxi Hospital, Taiyuan, 030032 China; 2 Institute of Urology, Beijing Chaoyang Hospital, Capital Medical University, Beijing, 100020 China; 3 Shanxi Bethune Hospital, Shanxi Academy of Medical Sciences, Tongji Shanxi Hospital, Third Hospital of Shanxi Medical University, Taiyuan, 030032 China; 4 Tongji Hospital, Tongji Medical College, Huazhong University of Science and Technology, Wuhan, 430030 China

**Keywords:** Cancer epidemiology, Endocrine cancer, Haematological cancer

## Abstract

While it is known that accurate evaluation of overall survival (OS) and disease-specific survival (DSS) for patients with primary adrenal lymphoma (PAL) can affect their prognosis, no stable and effective prediction model exists. This study aimed to develop prediction models to evaluate survival. This study enrolled 5448 patients with adrenal masses from the SEER Program. The influencing factors were selected using the least absolute shrinkage and selection operator regression model (LASSO) and Fine and Gray model (FGM). In addition, nomograms were constructed. Receiver operating characteristic curves and bootstrap self-sampling methods were used to verify the discrimination and consistency of the nomograms. The independent influencing factors for PAL survival were selected by LASSO and FGM, and three models were built: the OS, DSS, and FGS (DSS analysis by FGM) model. The areas under the curve and decision curve analyses indicated that the models were valid. This study developed survival prediction models to predict OS and DSS of patients with PAL. The FGS model was more accurate than the DSS model in the short term. Above all, these models should offer benefits to patients with PAL in terms of the treatment modality choice and survival evaluation.

## Introduction

Adrenal masses have become a worrisome threat among the global population, with a median incidence rate of approximately 3.0% (1.05–8.7%)^1^. Common pathological types include nonfunctional adrenal tumors, adrenocortical carcinomas, primary aldosteronism, primary adrenal lymphoma, Cushing’s syndrome, neuroendocrine tumor, pheochromocytoma and paraganglioma, myelolipomas, ganglioneuromas, and adrenal metastasis^2–4^.

Primary adrenal lymphoma (PAL) is extremely rare, accounting for approximately 5.8% of adrenal masses and 0.1% of all lymphomas, according to data from the Surveillance, Epidemiology, and End Results (SEER) Program. Some studies have shown that lymphoma can usually be found in lymph nodes, whereas PAL accounts for only approximately 1% of them; extranodal lymphomas are commonly found in the stomach and skin, while PAL accounts for 3% of extranodal lymphomas^5–7^. In addition, approximately 100 English-language publications on PAL have been published to date.

PAL refers to histopathologically confirmed lymphoma of the adrenal gland in the absence of previously diagnosed lymphoma at other sites or the co-occurrence of less prominent lesions at locations other than the adrenal gland^8,9^. PAL is a rare malignant tumor that seriously affects the quality of life and survival of patients. According to previous studies, the median overall survival (OS) is less than 3 years, and OS at 5 years is less than 20%^6^. Therefore, it is essential to determine the best patient regimen based on survival analysis.

The least absolute shrinkage and selection operator (LASSO)^10^, first proposed by Tibshirani, is an effective classifier for building machine-learning algorithms found in various classification or regression studies with good prediction results. A study dissecting the role of sphingolipid metabolism genes in osteosarcoma progression and microenvironment using LASSO to form a prognostic signature showed that the established model area under the curve (AUC) can reach 0.887^11^.

The Cox supportive hazards model (CoxPH) is a semiparametric regression model proposed in 1972 by Cox. With survival outcome and survival time as dependent variables, this model can simultaneously analyze the influence of many factors on survival, analyze data with censored survival time, and do not require the survival distribution of the estimated data. Because of these properties, this model has been widely used in medical follow-up studies and is one of the most widely used multivariate analysis methods for survival analysis. The Fine and Gray model (FGM), first proposed by Fine and Gray in 1999 for the distribution of a competing risk, can be used to exclude interference from competing events in survival analysis, resulting in a more accurate survival model^12,13^.

This study collected basic, clinical, and therapeutic data related to PAL from SEER, analyzed factors influencing OS and disease-specific survival (DSS), and established predicted prognosis models. We aimed to help clinicians make personalized treatment plans according to the individual conditions of patients to improve patient care and extend the expected survival period.

## Methods

### Data extraction

We extracted the data using SEER (SEER*Stat software released May 16, 2022, version 8.4.0.1), a database that includes cancer incidence and survival for more than one-third of the population of the United States. We obtained data from 11,168 patients with adrenal masses using the following three databases: Incidence-SEER Research Plus Data, 8 registries, Nov 2021 Sub [1975–2019], Incidence-SEER Research Plus Data, 12 registries, Nov 2021 Sub [1992–2019], and Incidence-SEER Research Plus Data, 17 registries, Nov 2021 Sub [2000–2019]). Screening was performed according to the following inclusion criteria: (1) mass originating in the adrenal gland (primary site code = C74.0/C74.1/C74.9); (2) precise pathological diagnosis; and (3) complete follow-up data. The exclusion criteria were as follows: (1) duplicate patient data, (2) incomplete data such as the age of the patient, and (3) missing follow-up data or loss of follow-up. A total of 5448 patients with adrenal masses were enrolled in the study. Lymphomas were selected based on histological codes (histology codes = 9590/3–9989/3). A total of 297 patients with adrenal lymphoma and 5151 with other adrenal masses were included (Supplementary Fig. S1).

### Clinicopathological characteristics

SEER data included 261 different variables, of which we selected the following: age, sex, race, side (laterality), tumor size (diameter), surgery, radiotherapy, chemotherapy, systemic therapy, marriage, income, residence, pathology, SEER stage, and Ann Arbor-Cotswolds (AAC) stage. For OS, death from any cause was defined as an event. DSS events were defined as death due to lymphoma.

### Statistical analysis

The data were processed using R 4.1.4 (Vienna Statistical Computing Foundation, Austria) and survival^14^ packages. The decision to use either the Shapiro–Wilk Normality Test or Kolmogorov–Smirnov Test was based on whether the sample size was less than 3000. As all continuous variables did not conform to a normal distribution, they were represented by the median (interquartile range). Categorical variables were expressed as frequencies and percentages (%). Survival between PAL and other adrenal tumors was analyzed using the Log-rank test and Kaplan–Meier (K–M) curves before and after propensity score matching (PSM). Patients with PAL were randomly divided into training and validation sets in a ratio of 7:3. Using the survival and glmnet packages, the univariate CoxPH model and LASSO were used to verify the independent factors influencing OS and DSS, respectively. The impact of each variable was analyzed using the Log-rank test and K–M curves. The nomogram model was then established according to the multivariate CoxPH, and the receiver operating characteristic (ROC) curve and the AUC were used to verify the prediction efficiency of the model. The bootstrap resampling method and calibration curve were used to evaluate the consistency of the model, and the net benefit to patients was assessed using clinical decision curve analysis (DCA). Statistical significance was set at P < 0.05 and marked with * in the tables.

### Ethics declarations

This study is not including human or animal subjects.

## Results

### Baseline characteristics

In total, 5448 patients were included in this study. Baseline patient characteristics are presented in Table 1. The age of the patients was 49.00 years (7.00, 65.00). Of these, 2682 (49.2%) were male and 2766 (50.8%) were female; 297 (5.5%) had lymphomas, and 5151 (94.5%) had other masses. The median OS was 28 months (95% confidence interval [CI] 18–41), and the 1-, 3-, 5-, and 10-year OS rates were 62.1%, 45.3%, 37.0%, and 28.0%, respectively. The median DSS was 43 months (95% CI 26–172), and the 1-, 3-, 5-, and 10-year DSS rates were 65.8%, 51.7%, 48.0%, and 43.7%, respectively.

**Table 1:** Baseline characteristics of the patients with adrenal tumors.

Variables	Total cohort (n = 5448)	Pathological types of adrenal tumors		F/χ^2^	P
		Lymphoma (n = 297)	Others (n = 5151)		
Age	49.00 [7.00,65.00]	72.00 [62.00,79.00]	47.00 [6.00,64.00]	18.422	< 0.001*
Sex					
Female	2766 (50.8)	107 (36.0)	2659 (51.6)	27.321	< 0.001*
Male	2682 (49.2)	190 (64.0)	2492 (48.4)		
Race					
White	4415 (81.0)	259 (87.2)	4156 (80.7)	18.953	< 0.001*
Black	543 (10.0)	8 (2.7)	535 (10.4)		
Asian or Pacific Islander	418 (7.7)	27 (9.1)	391 (7.6)		
American Indian/Alaska Native	41 (0.8)	2 (0.7)	39 (0.8)		
Unknown	31 (0.6)	1 (0.3)	30 (0.6)		
Side					
Unilateral	5026 (92.3)	201 (67.7)	4825 (93.7)	265.527	< 0.001*
Bilateral	422 (7.7)	96 (32.3)	326 (6.3)		
Surgery					
None	1359 (24.9)	213 (71.7)	1146 (22.2)	315.924	< 0.001*
Yes	2940 (54.0)	50 (16.8)	2890 (56.1)		
Unknown	1149 (21.1)	34 (11.4)	1115 (21.6)		
Radiotherapy					
Yes	1145 (21.0)	31 (10.4)	1114 (21.6)	21.179	< 0.001*
None/unknown	4303 (79.0)	266 (89.6)	4037 (78.4)		
Chemotherapy					
Yes	2527 (46.4)	220 (74.1)	2307 (44.8)	96.847	< 0.001*
None/unknown	2921 (53.6)	77 (25.9)	2844 (55.2)		
Systemic therapy					
None	1922 (35.3)	175 (58.9)	1747 (33.9)	55.366	< 0.001*
Yes	1068 (19.6)	22 (7.4)	1046 (20.3)		
Unknown	2458 (45.1)	100 (33.7)	2358 (45.8)		
Marriage					
Single (never married)	2324 (42.7)	32 (10.8)	2292 (44.5)	133.271	< 0.001*
Married	2210 (40.6)	197 (66.3)	2013 (39.1)		
Others/unknown	914 (16.8)	68 (22.9)	846 (16.4)		
Residence					
Urban	4274 (78.5)	233 (78.5)	4041 (78.5)	10.590	0.001*
Rural	604 (11.1)	53 (17.8)	551 (10.7)		
Unknown	570 (10.5)	11 (3.7)	559 (10.9)		
SEER stage					
Localized	1558 (28.6)	103 (34.7)	1455 (28.2)	27.493	< 0.001*
Regional	933 (17.1)	58 (19.5)	875 (17.0)		
Distant	2485 (45.6)	95 (32.0)	2390 (46.4)		
Unstaged	472 (8.7)	41 (13.8)	431 (8.4)		

Patients with PAL were older at diagnosis than those with other masses, had a higher incidence of male sex, were more frequently white, had a higher proportion of bilateral masses, and had lower SEER stage grades. Additionally, fewer patients underwent surgery and radiotherapy, whereas more patients underwent chemotherapy and systemic therapy.

A comparison of the OS and DSS of patients with PAL and others (Supplementary Fig. S2), the OS of PAL was lower than that of the others, while the difference in DSS between the two groups was not statistically significant. After PSM, both OS and DSS of PAL were higher than those of others, which might be because patients with PAL were older at diagnosis and had a higher proportion of bilateral masses than others, and PSM eliminated these effects.

Baseline data for PAL (Table 2) showed that patients who died were generally older, male, married, in poverty, living in rural areas, had bilateral masses, underwent treatment less frequently (including surgery, chemotherapy, radiotherapy, and systemic therapy), and had higher grades of SEER and AAC stages. According to the World Health Organization (WHO) classification criteria for lymphoma^15^, eight (2.7%) cases were Hodgkin’s lymphoma (HL), 280 (94.3%) were non-Hodgkin’s lymphoma (NHL), and nine (3%) were not otherwise specified (NOS). B-cell lymphoma (BCL) was predominant in NHL, of which the most common type was diffuse large B-cell lymphoma (DLBCL), with 223 cases (75.1%) (Table 3).

**Table 2:** Baseline characteristics of the patients with primary adrenal lymphomas.

Variables	Total cohort (n = 297)	Overall survival		F/χ^2^	P
		Dead (n = 198)	Alive (n = 99)		
Age	72.00 [62.00,79.00]	74.50 [66.25,81.00]	65.00 [54.50,73.00]	6.077	< 0.001*
Sex					
Female	107 (36.0)	63 (58.9)	44 (41.1)	4.565	0.033*
Male	190 (64.0)	135 (71.1)	55 (28.9)		
Race					
White	259 (87.2)	178 (68.7)	81 (31.3)	9.744	0.045*
Black	8 (2.7)	2 (25.0)	6 (75.0)		
Asian or Pacific Islander	27 (9.1)	15 (55.6)	12 (44.4)		
American Indian/Alaska Native	2 (0.7)	2 (100.0)	0 (0.0)		
Unknown	1 (0.3)	1 (100.0)	0 (0.0)		
Treatment					
None/unknown	55 (18.5)	42 (76.4)	13 (23.6)	2.856	0.091
Yes	242 (81.5)	156 (64.5)	86 (35.5)		
Surgery					
None	213 (71.7)	139 (65.3)	74 (34.7)	8.998	0.011*
Yes	50 (16.8)	29 (58.0)	21 (42.0)		
Unknown	34 (11.4)	30 (88.2)	4 (11.8)		
Radiotherapy					
Yes	31 (10.4)	21 (67.7)	10 (32.3)	0.018	0.893
None/unknown	266 (89.6)	177 (66.5)	89 (33.5)		
Chemotherapy					
Yes	220 (74.1)	142 (64.5)	78 (35.5)	1.718	0.190
None/unknown	77 (25.9)	56 (72.7)	21 (27.3)		
Systemic therapy					
None	175 (58.9)	103 (58.9)	72 (41.1)	28.191	< 0.001*
Yes	22 (7.4)	9 (40.9)	13 (59.1)		
Unknown	100 (33.7)	86 (86.0)	14 (14.0)		
Marriage					
Single (never married)	32 (10.8)	15 (46.9)	17 (53.1)	7.101	0.029*
Married	197 (66.3)	139 (70.6)	58 (29.4)		
Others/unknown	68 (22.9)	44 (64.7)	24 (35.3)		
Income					
Middle-class or affluent	279 (93.9)	181 (64.9)	98 (35.1)	6.778	0.034*
Poverty	12 (4.0)	11 (91.7)	1 (8.3)		
Unknown	6 (2.0)	6 (100.0)	0 (0.0)		
Residence					
Urban	233 (78.5)	151 (64.8)	82 (35.2)	5.900	0.052
Rural	53 (17.8)	36 (67.9)	17 (32.1)		
Unknown	11 (3.7)	11 (100.0)	0 (0.0)

**Table 3:** Oncological detail of primary adrenal lymphoma.

Variables	Total cohort (n = 297)	Overall survival		F/χ^2^	P
		Dead (n = 198)	Alive (n = 99)		
Side					
Unilateral	201 (67.7)	123 (61.2)	78 (38.8)	8.381	0.004*
Bilateral	96 (32.3)	75 (78.1)	21 (21.9)		
SEER stage					
Localized	103 (34.7)	65 (63.1)	38 (36.9)	9.662	0.022*
Regional	58 (19.5)	33 (56.9)	25 (43.1)		
Distant	95 (32.0)	65 (68.4)	30 (31.6)		
Unstaged	41 (13.8)	35 (85.4)	6 (14.6)		
AAC stage					
Stage I	89 (30.0)	66 (74.2)	23 (25.8)	30.495	< 0.001*
Stage II	54 (18.2)	34 (63.0)	20 (37.0)		
Stage III	13 (4.4)	10 (76.9)	3 (23.1)		
Stage IV	70 (23.6)	58 (82.9)	12 (17.1)		
Unstaged	71 (23.9)	30 (42.3)	41 (57.7)		
Pathology					
HL	8 (2.7)	4 (50.0)	4 (50.0)	24.371	0.041*
NSCHL	4 (1.3)	3 (75.0)	1 (25.0)		
MCCHL	1 (0.3)	0 (0.0)	1 (100.0)		
NOS	3 (1.0)	1 (33.3)	2 (66.7)		
NHL	280 (94.3)	185 (66.1)	95 (33.9)		
BCL	254 (85.5)	166 (65.4)	88 (34.6)		
DLBCL	223 (75.1)	146 (65.5)	77 (34.5)		
FL	11 (3.7)	5 (45.5)	6 (54.5)		
PCN	6 (2.0)	6 (100.0)	0 (0.0)		
BL	4 (1.3)	2 (50.0)	2 (50.0)		
CLL/SLL	4 (1.3)	4 (100.0)	0 (0.0)		
MALT	4 (1.3)	1 (25.0)	3 (75.0)		
MCL	2 (0.7)	2 (100.0)	0 (0.0)		
NKTCL	8 (2.7)	8 (100.0)	0 (0.0)		
PTCL	6 (2.0)	6 (100.0)	0 (0.0)		
ALCL	1 (0.3)	1 (100.0)	0 (0.0)		
ENKTCL	1 (0.3)	1 (100.0)	0 (0.0)		
NOS	18 (6.1)	11 (61.1)	7 (38.9)		
NOS	9 (3.0)	9 (100.0)	0 (0.0)		

*AAC stage* Ann Arbor-Cotswolds stage, *HL* Hodgkin lymphoma, *NSCHL* nodular sclerosing classical Hodgkin lymphoma, *MCCHL* mixed cellularity classical Hodgkin lymphoma, *NOS* not otherwise specified, *NHL* non-Hodgkin lymphoma, *BCL* B-cell lymphoma, *DLBCL* diffuse large B-cell lymphoma, *FL* follicular lymphoma, *PCN* plasmacytoma, *BL* Burkitt lymphoma, *CLL/SLL* chronic lymphocytic leukemia/small lymphocytic lymphoma, *MALT* mucosa-associated lymphoid tissue lymphoma, *MCL* mantle cell lymphoma, *NKTCL* NK/T-cell lymphoma, *PTCL* peripheral T-cell lymphoma, *ALCL* anaplastic large cell lymphoma, *ENKTCL* extranodal NK-/T-cell lymphoma, nasal type.

### Univariate analysis

Univariable CoxPH analysis suggested that the factors influencing OS (Supplementary Figs. S3 and S4a–e) were: age (odds ratio [OR]: 1.053, 95% CI 1.038–1.067, P < 0.001), sex (OR: 1.469, 95% CI 1.086–1.987, P = 0.013), race (American Indian or Alaska Native/White) (OR: 10.186, 95% CI 2.453–42.306, P = 0.001), side (bilateral/unilateral) (OR: 1.886, 95% CI 1.412–2.520, P < 0.001), treatment (OR: 0.668, 95% CI 0.475–0.941, P = 0.021), surgery (OR: 0.527, 95% CI 0.350–0.793, P = 0.002), systemic therapy (OR: 0.470, 95% CI 0.237–0.931, P = 0.030), income (poverty/middle class or affluent [MCA]) (OR: 2.048, 95% CI 1.113–3.769, P = 0.021), and AAC stage (stage IV/stage I) (OR: 1.475, 95% CI 1.034–2.103, P = 0.032).

Those influencing DSS (Supplementary Figs. S4f–j and S5) were: age (OR: 1.048, 95% CI 1.032–1.065, P < 0.001), side (bilateral/unilateral) (OR: 2.350, 95% CI 1.693–3.261, P < 0.001), surgery (OR: 0.416, 95% CI 0.242–0.716, P = 0.002), SEER stage (distant/localized) (OR: 1.599, 95% CI 1.061–2.411, P = 0.025), and AAC stage (stage IV/stage I) (OR: 1.638, 95% CI 1.081–2.480, P = 0.020).

Univariate FGM (Supplementary Fig. S6) suggested that the DSS analysis by FGM (FGS) was as follows: age (F = 25.379, P < 0.001), side (F = 27.529, P < 0.001), surgery (F = 9.924, P = 0.002), and SEER stage (F = 6.074, P = 0.048).

### LASSO

We converted the multi-categorical variables into binary-categorical variables by using dummy variables. Table 4 presents the final variable assignments. A tenfold cross-validation was performed for all variables using LASSO CoxPH regression with the log (lambda) value of the harmonic parameter. The partial likelihood deviance of the model changed with a change in lambda. The corresponding number of variables filtered by the OS model is shown in Supplementary Fig. S7a, whereas that of the DSS is shown in Supplementary Fig. S7b. We constructed an influencing factor classifier using the LASSO regression model (Supplementary Fig. S7c,d). After LASSO, 11 factors influencing OS were selected, including age, sex, side, surgery, systemic, income, pathology, SEER stage = regional, SEER stage = distant, AAC stage = stage II, and AAC stage = stage IV. Six influencing factors of DSS were selected: age, side, surgery, income, SEER stage (distant), and AAC stage (stage IV) (Table 5).

**Table 4:** Variable assignments.

Variable	Risk factors	Assignments
X_1_	Age	Continuous variable
X_2_	Sex	Female = 0, male = 1
X_3_	Side	Unilateral = 0, bilateral = 1
X_4_	Treatment	No = 0, yes = 1
X_5_	Surgery	No = 0, yes = 1
X_6_	Radiation	No = 0, yes = 1
X_7_	Chemotherapy	No = 0, yes = 1
X_8_	Systemic	No = 0, yes = 1
X_9_	Income	MCA = 0, poverty = 1
X_10_	Pathology	HL = 0, NHL = 1
X_11_	Race = white	No = 0, yes = 1
X_12_	Race = black	No = 0, yes = 1
X_13_	Race = Asian or Pacific islander	No = 0, yes = 1
X_14_	Race = American Indian/Alaska native	No = 0, yes = 1
X_15_	Marriage = single (never married)	No = 0, yes = 1
X_16_	Marriage = married	No = 0, yes = 1
X_17_	SEER stage = localized	No = 0, yes = 1
X_18_	SEER stage = regional	No = 0, yes = 1
X_19_	SEER stage = distant	No = 0, yes = 1
X_20_	AAC stage = stage I	No = 0, yes = 1
X_21_	AAC stage = stage II	No = 0, yes = 1
X_22_	AAC stage = stage III	No = 0, yes = 1
X_23_	AAC stage = stage IV	No = 0, yes = 1

**Table 5:** Risk factors selected by LASSO.

Variable	Risk factors	Coefficient	Odds ratio
Overall survival			
X_1_	Age	0.046	1.078
X_2_	Sex	0.075	1.399
X_3_	Side	0.336	0.850
X_5_	Surgery	− 0.163	0.922
X_8_	Systemic therapy	− 0.082	2.987
X_9_	Income	1.094	1.331
X_10_	Pathology	0.286	0.949
X_18_	SEER stage = regional	− 0.052	1.002
X_19_	SEER stage = distant	0.002	0.999
X_21_	AAC stage = stage II	− 0.001	1.082
X_23_	AAC stage = stage IV	0.079	1.078
Disease-specific survival			
X_1_	Age	0.039	1.039
X_3_	Side	0.226	1.253
X_5_	Surgery	− 0.111	0.895
X_9_	Income	0.789	2.202
X_19_	SEER stage = distant	0.050	1.051

### Nomogram

**Figure 1:**
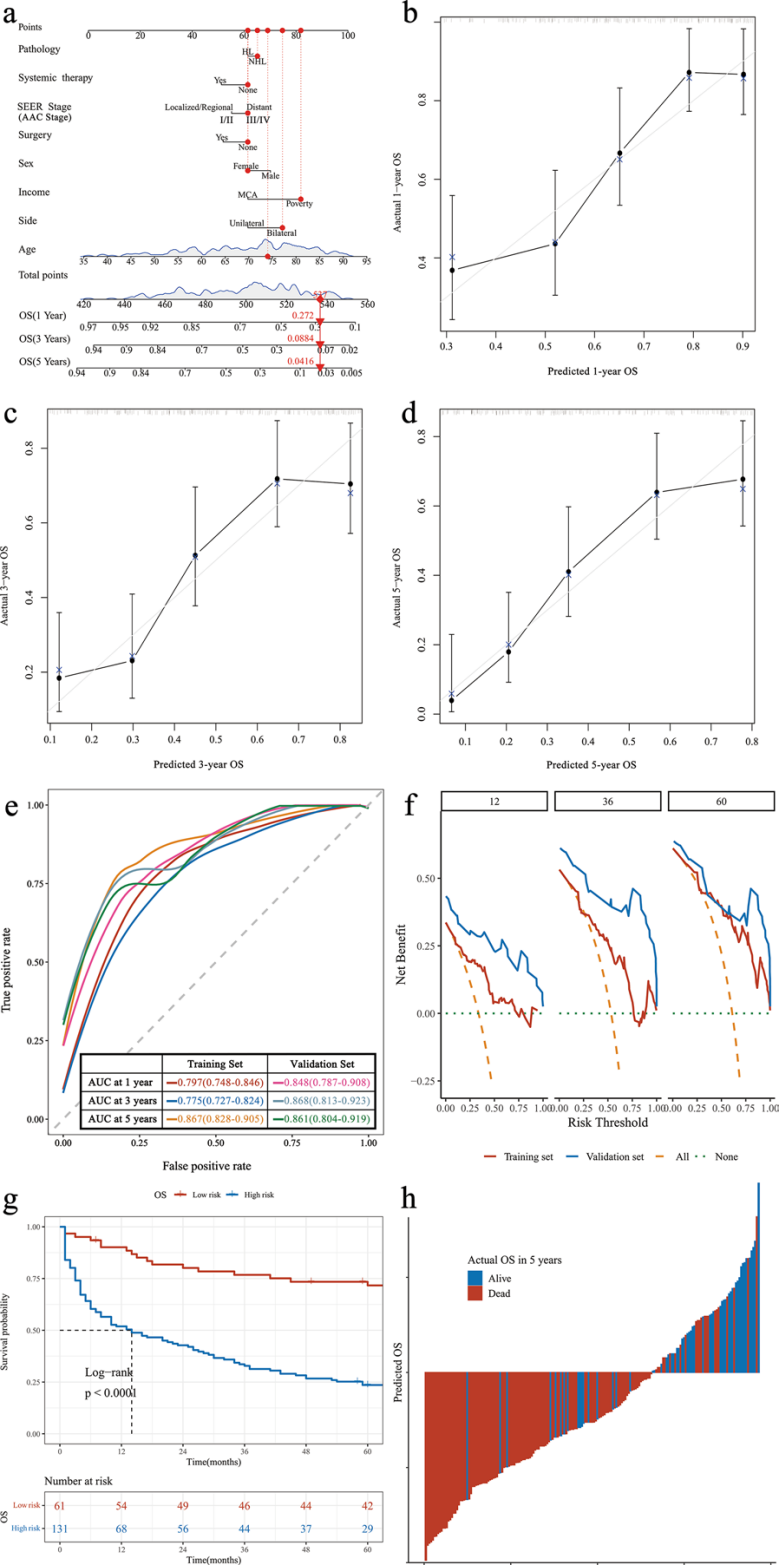
Nomogram of OS in patients with PAL. (**A**) Nomogram; (**B**) calibration curves in 1 year; (**C**) calibration curves in 3 years; (**D**) calibration curves in 5 years; (**E**) ROC curves of the nomogram in the training and validation sets. (**F**) Clinical decision curve analysis of prediction model. (**G**) Kaplan Meier curves for predicting risk subgroups according to the nomogram; (**H**) the calculated risk scores for each patient within the combined training and validation sets.

**Figure 2:**
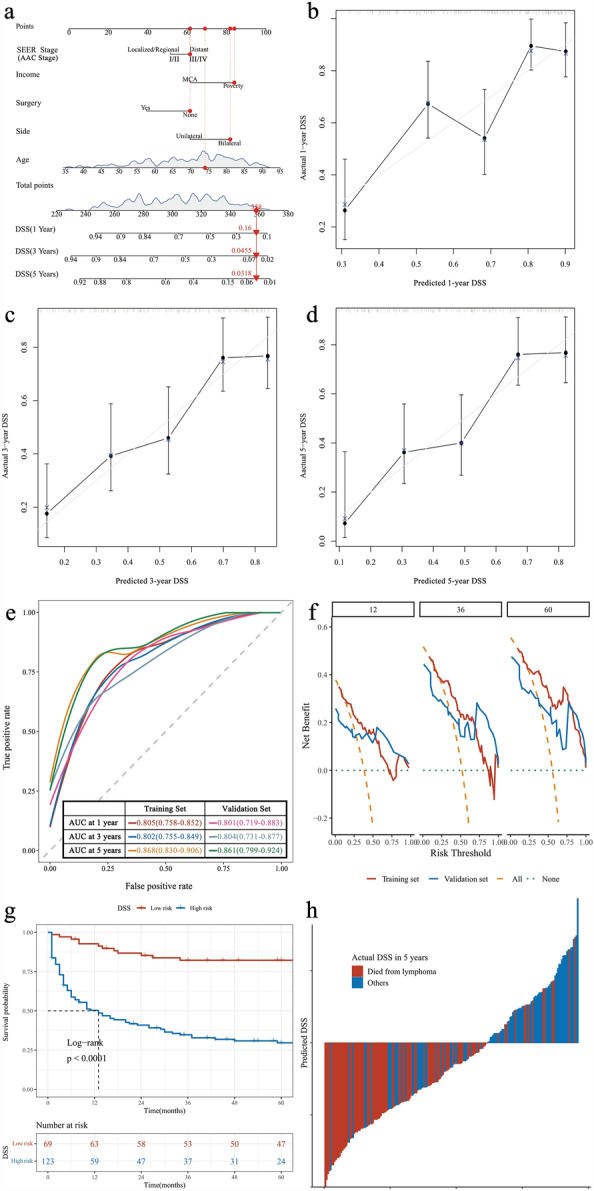
Nomogram of DSS in patients with PAL. (**A**) Nomogram; (**B**) calibration curves in 1 year; (**C**) calibration curves in 3 years; (**D**) calibration curves in 5 years; (**E**) ROC curves of the nomogram in the training and validation sets. (**F**) Clinical decision curve analysis of prediction model. (**G**) Kaplan Meier curves for predicting risk subgroups according to the nomogram; (**H**) the calculated risk scores for each patient within the combined training and validation sets.

**Figure 3:**
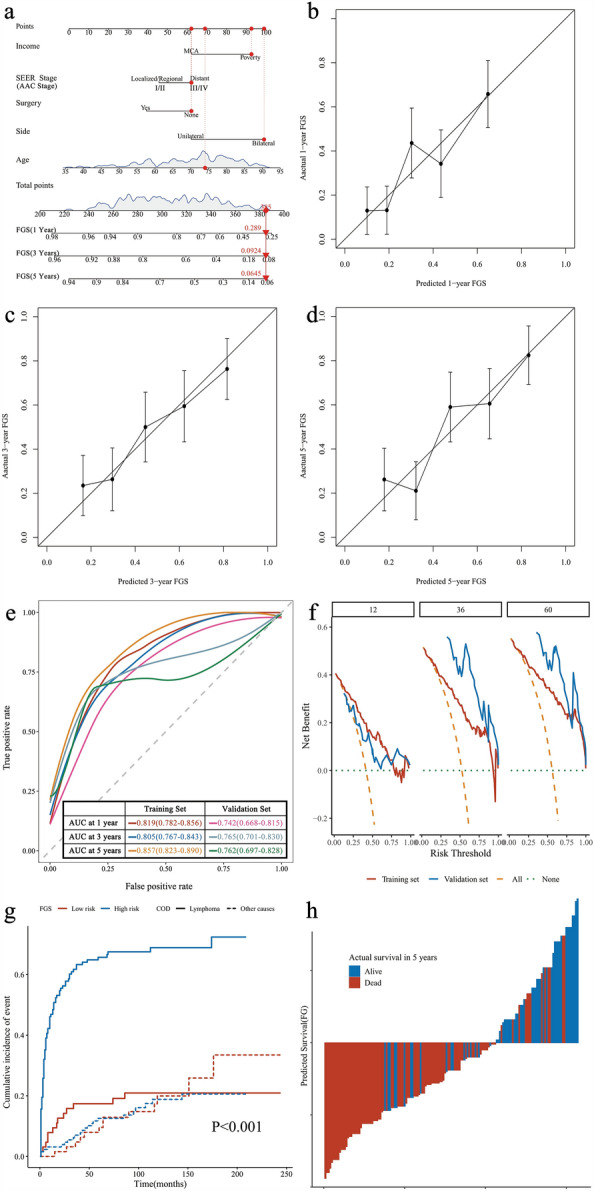
Nomogram of survival according to the Fine and Gray model (FGS) in patients with PAL. (**A**) Nomogram; (**B**) calibration curves in 1 year; (**C**) calibration curves in 3 years; (**D**) calibration curves in 5 years; (**E**) ROC curves of the nomogram in the training and validation sets. (**F**) Clinical decision curve analysis of prediction model. (**G**) Fine and Gray model for predicting risk subgroups according to the nomogram; (**H**) the calculated risk scores for each patient within the combined training and validation sets.

The models (OS, DSS, and FGS models) built by LASSO and FGM are represented by nomograms (Figs. 1a, 2a, and 3a). We utilized the "regplot" package in R to generate nomograms, with the "center" parameter set to "T". The nomograms were created based on the results of the LASSO and multivariable CoxPH. The principle underlying nomograms involves converting the variable with the most extensive product of the coefficient and variable span (Age) to 100 and converting other variables to the same ratio. This approach enables simplified calculations while maintaining the prediction accuracy. For example, in Fig. 1a, the maximum and minimum values of Age (91 and 36) were collapsed to 100 and 0, respectively; HL and NHL in Pathology corresponded to 61 and 65, respectively; the presence or absence of Systemic therapy corresponded to 51 and 61, respectively; Localized/Regional and Distant in SEER Stage corresponded to 55 and 61, respectively; the presence or absence of Surgery corresponded to 61 and 52, respectively; Female and Male corresponded to 61 and 70, respectively; MCA and Poverty corresponded to 61 and 82, respectively; Unilateral and Bilateral corresponded to 61 and 75, respectively. The method of nomograms is based on the score corresponding to each predictor of a patient, summed to calculate the total score, and the risk value corresponding to the score of the total points is the probability of an event for a patient (dead for OS, dead from PAL for DSS and FGS)^16^. A patient with ID 50031326 is used as an example. This patient was a 73-year-old female living in poverty with bilateral NHL without surgery or systemic therapy. The SEER stage was distinct, and the AAC stage was stage IV. Thus, the scores corresponding to each index of the OS analysis were 65, 61, 61, 61, 61, 82, 75, and 71; the total score was 537; and the corresponding 1-, 3-, and 5-year OS rates were 27.2%, 8.84%, and 4.16%, respectively. The scores corresponding to each analysis index of DSS were 61, 83, 61, 82, and 71; the total score was 358; and the corresponding 1-, 3-, and 5-year DSS were 16.00%, 4.55%, and 3.18%, respectively. The scores corresponding to each FGS index were 93, 61, 61, 99, and 71, with a total score of 385, and the corresponding 1-, 3-, and 5-year FGS were 28.90%, 9.24%, and 6.45%, respectively.

### Validation and performance of nomogram

The C-index of the predicted model for OS was 0.727 (95% CI 0.680–0.774), and after 1000 resampling internal validations, the calibration curve fitted well with the ideal curve, indicating that the predicted probability of the model had good uniformity and stability compared to the actual model (Fig. 1b–d). ROC curves were drawn according to the model-fitting results. The AUC of the 1-year OS were 0.797 (95% CI 0.748–0.846) and 0.848 (95% CI 0.787–0.908) for the training and validation sets, respectively. The AUC values of 3-year OS were 0.775 (95% CI 0.727–0.824) and 0.868 (95% CI 0.813–0.923). The AUC values of 5-year OS were 0.867 (95% CI 0.828–0.905) and 0.861 (95% CI 0.804–0.919). These results indicate that the model has high predictive ability (Fig. 1e). DCA according to the model showed that the model could improve the net benefit to patients by up to 18%, 33%, and 45% at 1, 3, and 5 years, respectively (Fig. 1f). The subgroup was divided according to the risk predicted by the model, and the median OS was 237 months (95% CI:151-not reached [NR]) for the low-risk group and 14 months (95% CI 8–26) for the high-risk group (Fig. 1g). In addition, the model had a sensitivity of 0.832, specificity of 0.696, and Youden Index of 0.528, resulting in a high prediction accuracy (Fig. 1h).

The C-index of the predicted model for DSS was 0.732 (95% CI 0.679–0.785), and after 1000 resampling internal validations, the calibration curve fitted well with the ideal curve, indicating that the predicted probability of the model had good uniformity and stability with the actual probability (Fig. 2b–d). ROC curves were drawn according to the model-fitting results. The AUC of the 1-year DSS were 0.805 (95% CI 0.758–0.852) and 0.801 (95% CI 0.719–0.883) for the training and validation sets, respectively. The AUC of 3-year DSS were 0.802 (95% CI 0.755–0.849) and 0.804 (95% CI 0.831–0.877). The AUC of 5-year DSS were 0.868 (95% CI 0.830–0.906) and 0.861 (95% CI 0.799–0.924). These results indicate that the model has high predictive ability (Fig. 2e). DCA according to the model showed that the model could improve the net benefit to patients by up to 18%, 31%, and 40% at 1, 3, and 5 years, respectively (Fig. 2f). The subgroup was divided according to the risk predicted by the model, and the median DSS was NR (95% CI NR–NR) for the low-risk group and 13 months (95% CI 7–26) for the high-risk group (Fig. 2g). In addition, the model had a sensitivity of 0.861, specificity of 0.604, and Youden Index of 0.465, resulting in a high prediction accuracy (Fig. 2h).

The C-index of the predicted model for FGS was 0.727 (95% CI 0.674–0.780), and after 1000 resampling internal validations, the calibration curve fitted the ideal curve well, indicating that the predicted probability of the model was in good agreement with the actual probability (Fig. 3b–d). ROC curves were drawn according to the model-fitting results. The AUC values of the 1-year FGS were 0.819 (95% CI 0.782–0.856) and 0.742 (95% CI 0.668–0.815) for the training and validation sets, respectively. The AUC values of 3-year FGS were 0.805 (95% CI 0.767–0.843) and 0.765 (95% CI 0.701–0.830). The AUC values of 5-year FGS were 0.857 (95% CI 0.823–0.890) and 0.762 (95% CI 0.697–0.828). These results indicate that the model has a high predictive ability (Fig. 3e). DCA, according to the model, showed that the model could improve the net benefit of patients by approximately 20%, 32%, and 38% at 1, 3, and 5 years, respectively (Fig. 3f). The subgroup was divided according to the risk predicted by the model, and the median FGS was NR (95% CI NR–NR) for the low-risk group and 8 months (95% CI 5–14) for the high-risk group (Fig. 3g). In addition, there was a significant difference in survival between patients who died due to PAL in the low- and high-risk groups (F = 39.616, P < 0.001), but not in those who died due to other causes (F = 0.192, P = 0.661). Moreover, the model had a sensitivity of 0.871, specificity of 0.679, and Youden index of 0.550, indicating a high prediction accuracy (Fig. 3h).

## Discussion

PAL, a rare malignant mass, is a hot topic in the medical community. The prognosis of the patients was poor, the median OS was less than 3 years, and the 10-year OS was less than 30%. At present, most English language publications are case reports of DLBCL, and survival analysis is limited^6^.

In recent years, analyses based on large public databases have become a trend in the medical field^17^. Public database analysis has several advantages. First, it provides shared data resources, saving time and costs in collecting and generating data. Second, public databases validated and verified the data, ensuring the reliability of the research results. By analyzing these databases, new research directions and associations can be discovered. It also promotes collaboration and data sharing among researchers, thereby accelerating research progress. Additionally, public database analysis saves research costs and time, and improves research efficiency^13,18^.

This study compared the survival of PAL with that of other adrenal masses using PSM based on SEER data. Factors influencing the OS and DSS of patients were analyzed using Cox regression, and the prognosis of patients was analyzed using LASSO and FGM to construct a prediction model of OS, including age, sex, side, surgery, systemic therapy, income, pathology, and AAC stage (SEER stage), and compared with a DSS model including age, side, surgery, income, and AAC stage. The accuracy of the visible model was high based on ten-fold cross-validation.

Age is a significant factor that influences the prognosis and survival of almost all patients with carcinomas. Older patients tend to have poor nutritional status and tolerability. In contrast, masses at diagnosis tended to be at a higher stage^19–21^. There is some controversy regarding the prognosis of carcinoma according to sex. Some researchers suggest that men may have more risky lifestyles (e.g., smoking) and that men and women differ in hormone levels, leading to a worse prognosis^22–24^. A systematic review and meta-analysis of cancer immunotherapy efficacy and patient sex showed that men and women have different sensitivities to immune checkpoint inhibitors; thus, their prognosis may differ^25^. A review found that poor individuals had a lower OS than MCA individuals. This is not only due to higher rates of smoking, obesity, and substance abuse, but also because of unequal access to technological innovation, increased geographic isolation by income, reduced economic mobility, mass incarceration, and increased healthcare costs^26^.

In addition to the basic characteristics mentioned above, oncological characteristics also affect patient survival. Most extranodal lymphomas are NHL, whereas HL tends to progress to extranodal lymphoma with very few extreme malignancies. Therefore, among the pathological types of PAL, HL has a significantly worse prognosis than NHL^27^. Patients with bilateral masses tended to have lower survival rates, which is similar to other carcinomas and consistent with many reported results^6,7,28,29^. AAC stage is a lymphoma stage classification system approved by the WHO for both HL and NHL. A higher grade represents a greater extent of infiltration, and more sites are involved^30^. Based on the extent of invasion, the SEER stage classifies masses into three grades: localized, regional, and distant. The two classification systems were consistent.

Treatment modality similarly affected prognosis. In this study, treated patients had a much better prognosis than untreated patients, showing that surgery and systemic therapy are particularly important. Surgery included local tumor excision, simple/partial surgery, total surgery, and radial surgical removal of the primary site. Early surgery can improve patient survival. In addition, for adrenal masses, minimally invasive surgery can be of benefit^31–34^. Systemic therapy is a form of psychotherapy that focuses on how individuals’ personal relationships, behavioral patterns, and life choices relate to the problems they face in their lives. This reduces the unintended risk to the patient and significantly improves OS; however, the impact of systemic therapy on DSS is not obvious. Radiotherapy does not seem to play a role in PAL prognosis. A clinical trial comparing chemotherapy with or without radiotherapy in DLBCL showed that the OS and DSS did not differ between the two groups^35^. In this study, chemotherapy appeared to improve the median OS (28 months [95% CI 18–41] vs. 23 months [95% CI 9–53]), but the difference was not significant. In the course of further analysis, it was found that patients with DLBCL had significantly improved OS after chemotherapy (27 months [95% CI 17, 50] vs. 9 months [95% CI 2, 53], P = 0.033). DLBCL was more sensitive to chemotherapy than other tumors, a result similar to those of other studies^6^.

Currently, only a few survival studies have been conducted on PAL. The innovation of this study is that the factors influencing survival in PAL were analyzed using LASSO, and FGM excluded the interference of other events. The prediction models for OS and DSS were established and validated, making this model more meaningful. According to the predicted prognostic model designed by the FGM, the predicted FGS was not statistically different from the DSS model, and the predicted survival accuracy was higher in the short term (less than 3 years). Therefore, it is more accurate to exclude the influence of death from other factors.

The limitations of this study are as follows: (1) as a rare malignant mass, the number of cases was small; (2) the period of cases was extensive, and the living environments and treatment conditions experienced by patients in different periods varied; and (3) LASSO and FGM were adopted in this study, which will be compared with other machine learning algorithms in a subsequent study, leading to the best model.

In conclusion, age, sex, side, surgery, systemic therapy, income, pathology, and AAC stage (SEER stage) affected OS in patients with PAL. Age, side, surgery, income, and AAC stage affected DSS. The prediction models for OS and DSS built using LASSO and FGM showed good predictive performance. The model created by FGM is more accurate in the short term (less than 3 years). Using this model, the survival expectations of patients with PAL can be effectively evaluated, enabling clinicians to individualize the design of treatment regimens, improve the expected survival of patients, and further benefit patients.

### Supplementary Information

Supplementary Figures.

## Data Availability

Data supporting reported results can be found through SEER (SEER*Stat software released May 16, 2022, version 8.4.0.1). (https://seer.cancer.gov/seerstat/).
