# Supplementary material for: Development and validation of nomograms to predict survival of primary adrenal lymphoma: a population-based retrospective study
Source: Sci Rep. 2023 Sep 2;13:14428. doi: 10.1038/s41598-023-41839-2 (PMC10475110; doi:10.1038/s41598-023-41839-2)
Supplement: Supplementary file 1 — Supplementary Figures. [file 41598_2023_41839_MOESM1_ESM.pdf]

Supplementary Information

Supplementary Figure S1. Study flowchart

Supplementary Figure S2. Survival analysis before and after propensity score matching (PSM). (a. Overall survival [OS] before PSM; b. OS after PSM; c. disease-specific survival (DSS) before PSM; d. DSS after PSM.)

Supplementary Figure S3. Kaplan–Meier curves of OS for patients based on: (a) Age; (b) Sex; (c) Race; (d) Marriage; (e) Income; (f) Residence; (g) SEER stage; (h) AAC stage.

Supplementary Figure S4. Kaplan–Meier curves of OS and DSS for patients based on treatment.
(a. OS based on treatment; b. OS based on surgery [Su]; c. OS based on radiotherapy [R]; d. OS based on chemotherapy [C]; e. OS based on Systemic therapy [Sy]; f. DSS based on treatment; g. DSS based on Su; h. DSS based on R; i. DSS based on C; j. DSS based on Sy.)

Supplementary Figure S5. Kaplan–Meier curves of DSS for patients based on: (a) Age; (b) Sex; (c) Race; (d) Marriage; (e) Income; (f) Residence; (g) SEER stage; (h) AAC stage.

Supplementary Figure S6. Fine-Grey Model for patients based on: (a) Age; (b) Sex; (c) Race; (d) Marriage; (e) Income; (f) Residence; (g) SEER stage; (h) AAC stage.

Supplementary Figure S7. LASSO regression. (a. The 10- fold cross-validation results of OS; b. The 10- fold cross-validation results of DSS; c. LASSO coefficient profiles of the 23 variables of OS; d. LASSO coefficient profiles of the 23 variables of DSS.)

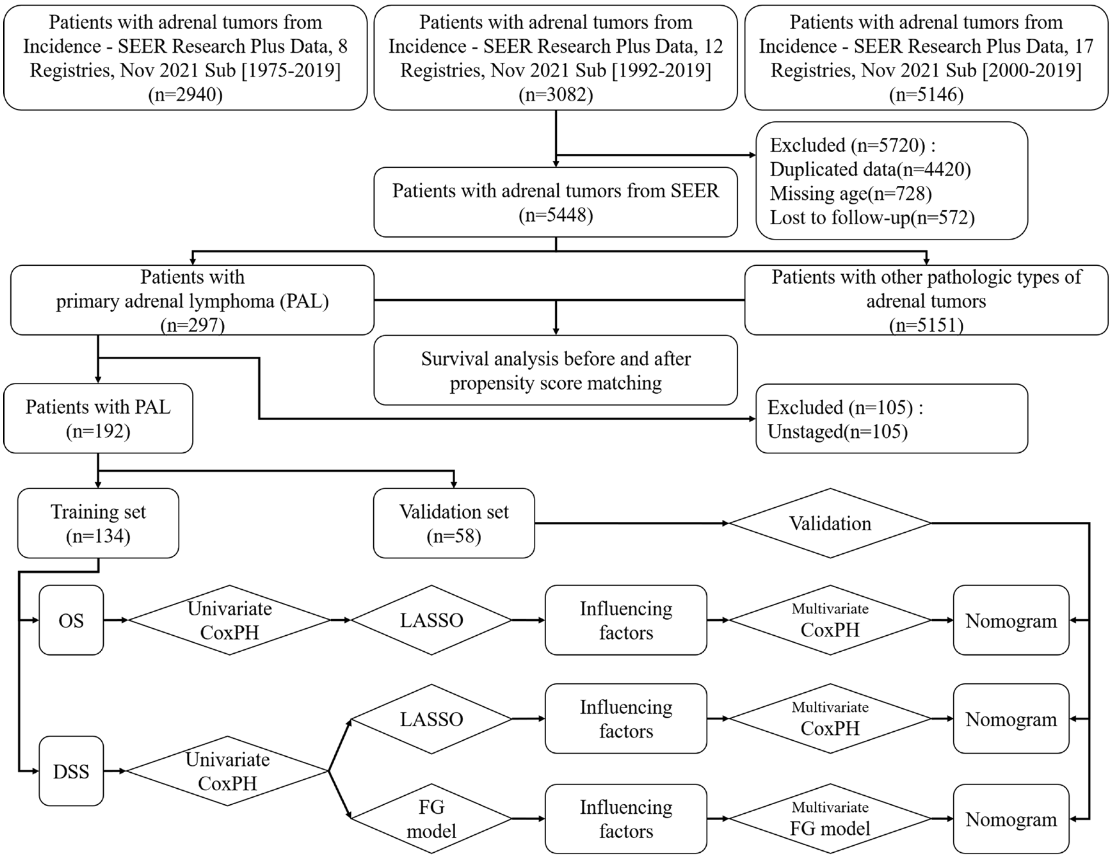

Supplementary Figure S1. Study flowchart

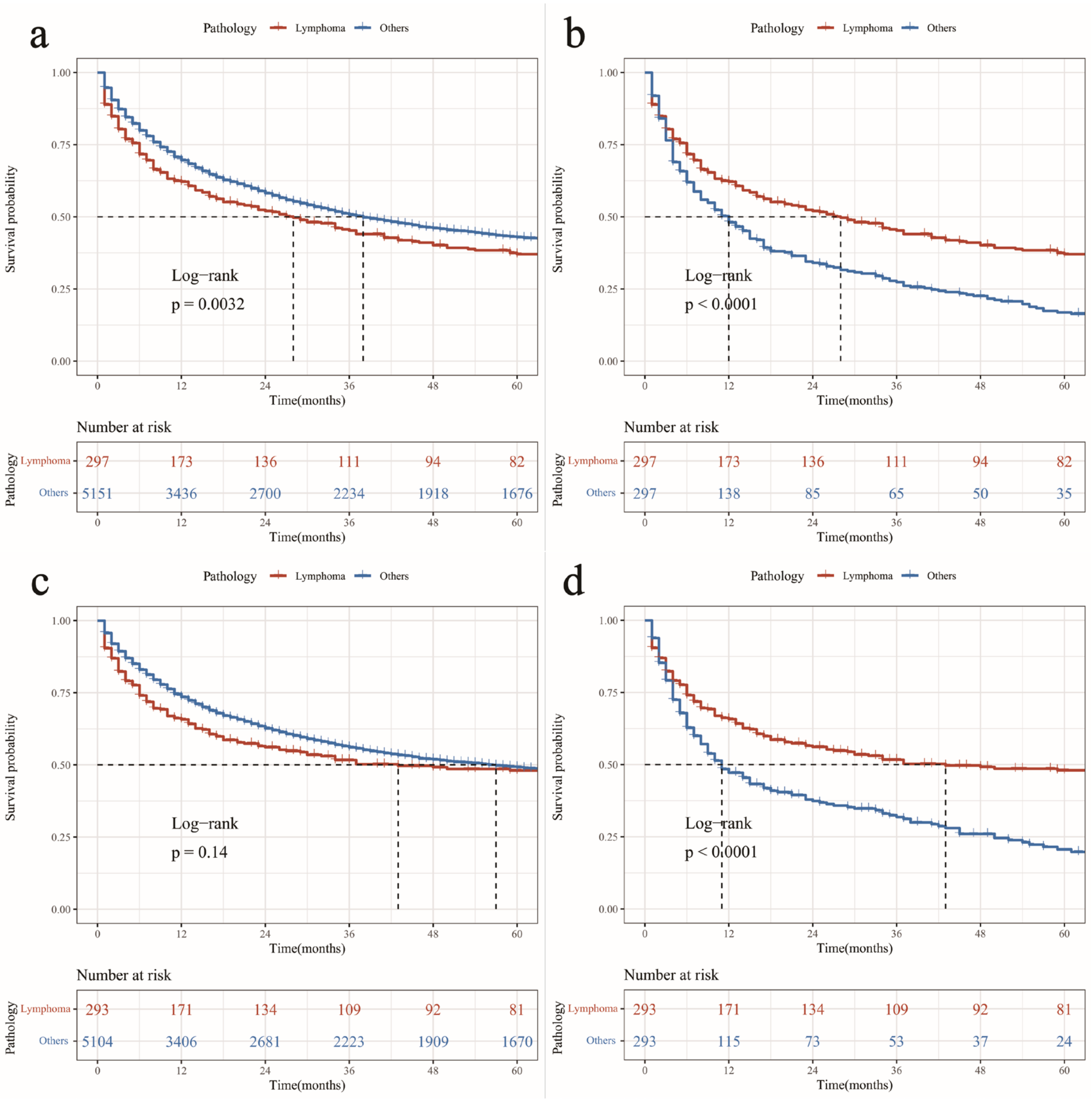

Supplementary Figure S2. Survival analysis before and after propensity score matching (PSM).

(a. Overall survival [OS] before PSM; b. OS after PSM;
c. disease-specific survival (DSS) before PSM; d. DSS after PSM.)

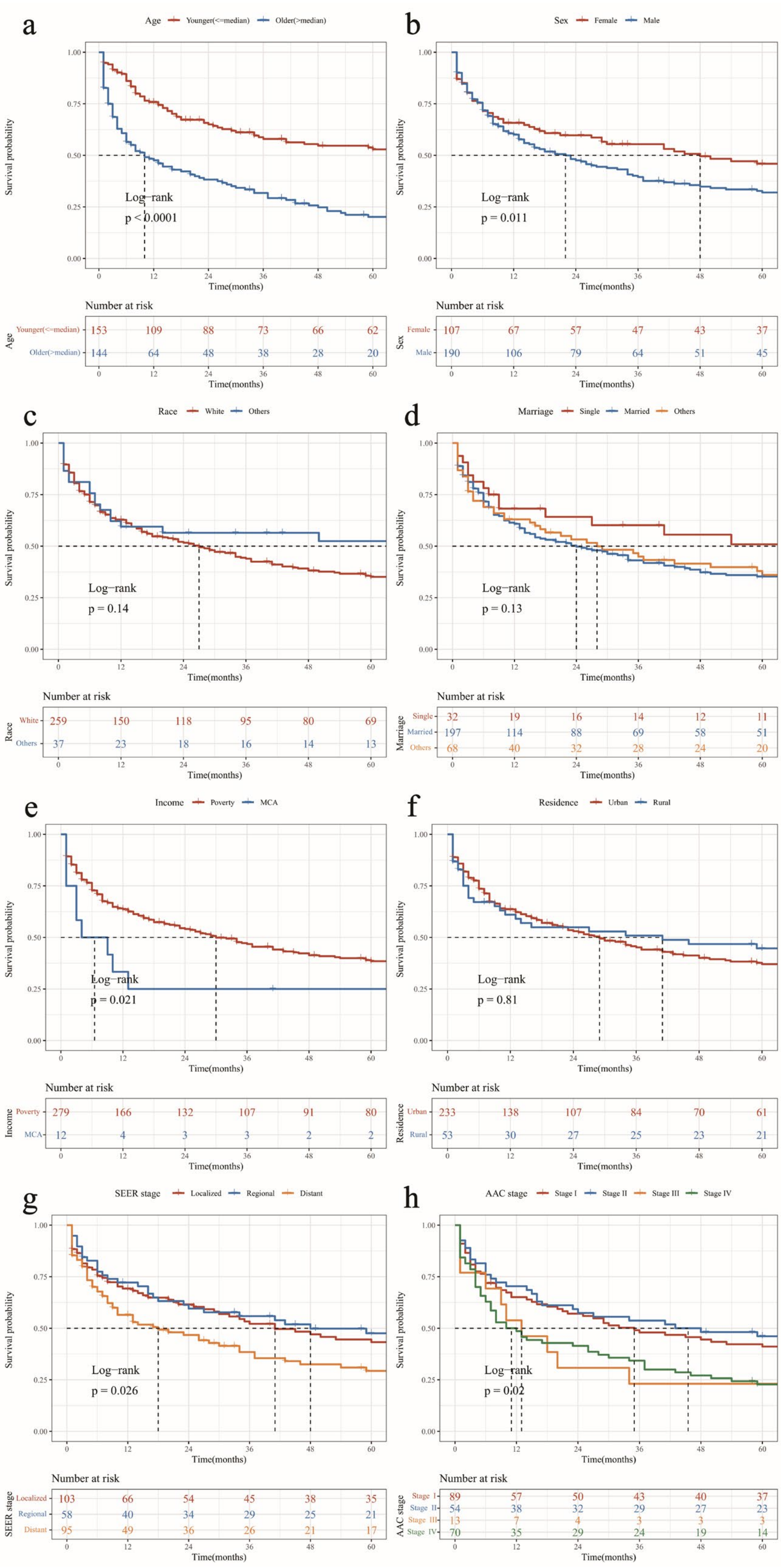

Supplementary

Figure S3.

Kaplan–Meier curves of OS for patients based on:

(a) Age;
(b) Sex;
(c) Race;
(d) Marriage;
(e) Income;
(f) Residence;
(g) SEER stage;
(h) AAC stage.

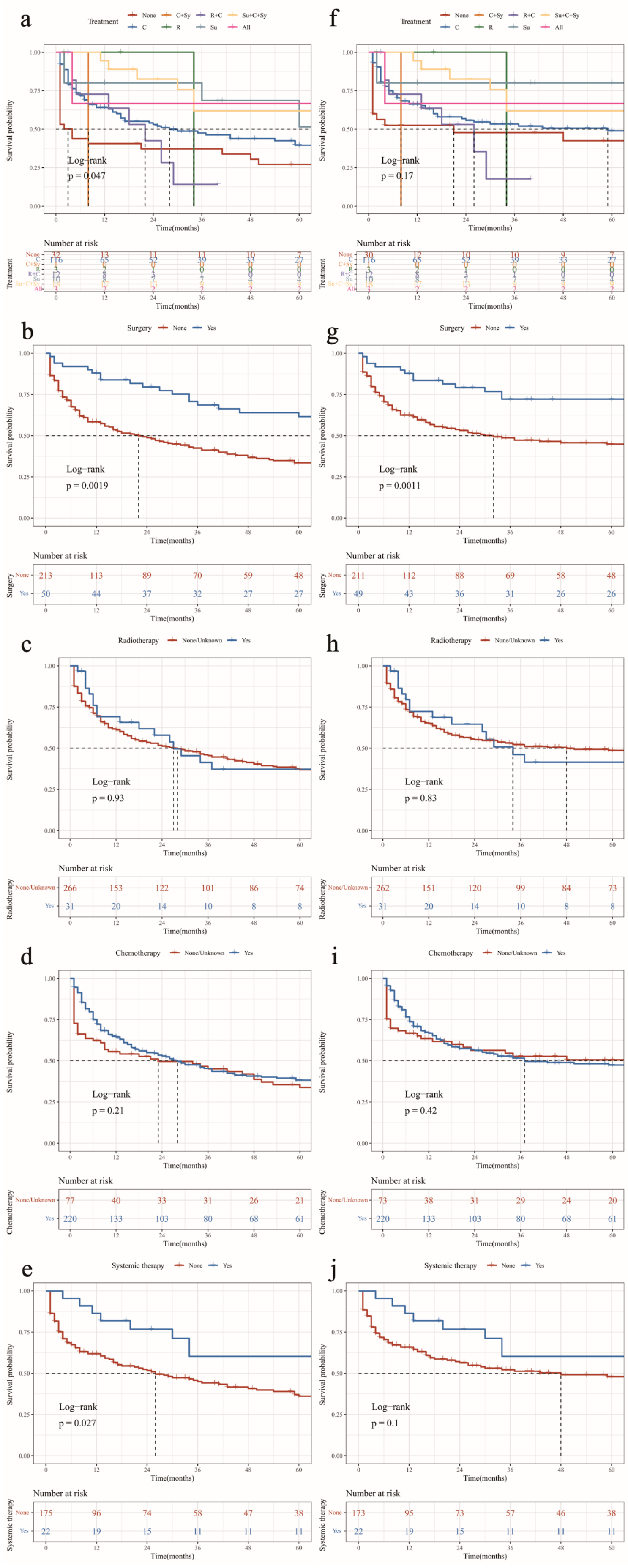

Supplementary

Figure S4. Kaplan–Meier curves of OS and DSS for patients based on treatment. (a. OS based on treatment; b. OS based on surgery [Su]; c. OS based on radiotherapy [R]; d. OS based on chemotherapy [C]; e. OS based on Systemic therapy [Sy]; f. DSS based on treatment; g. DSS based on Su; h. DSS based on R; i. DSS based on C; j. DSS based on Sy.)

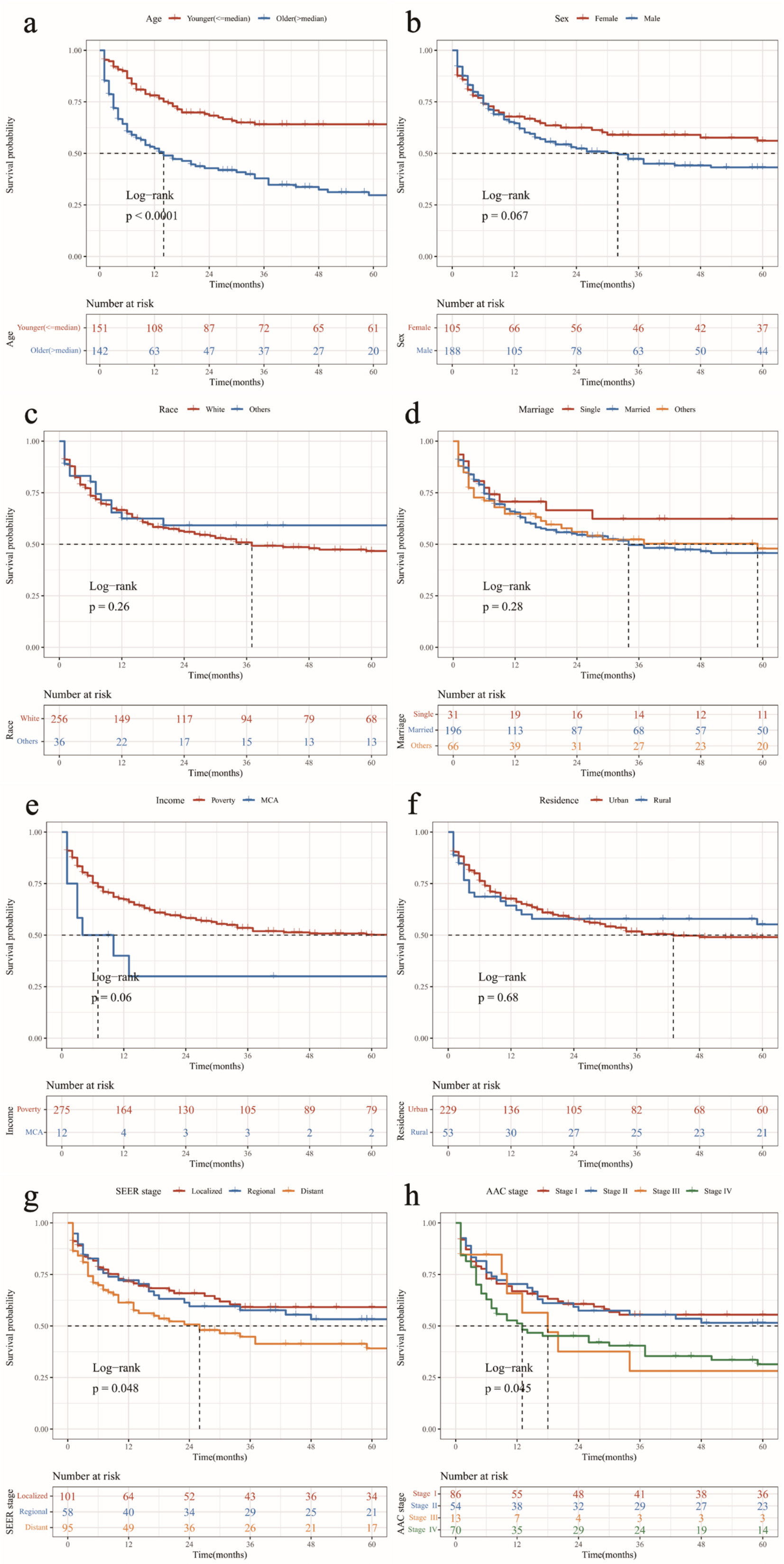

Supplementary

Figure S5.

Kaplan–Meier curves of DSS for patients based on:
(a) Age;
(b) Sex;
(c) Race;
(d) Marriage;
(e) Income;
(f) Residence;
(g) SEER stage;
(h) AAC stage.

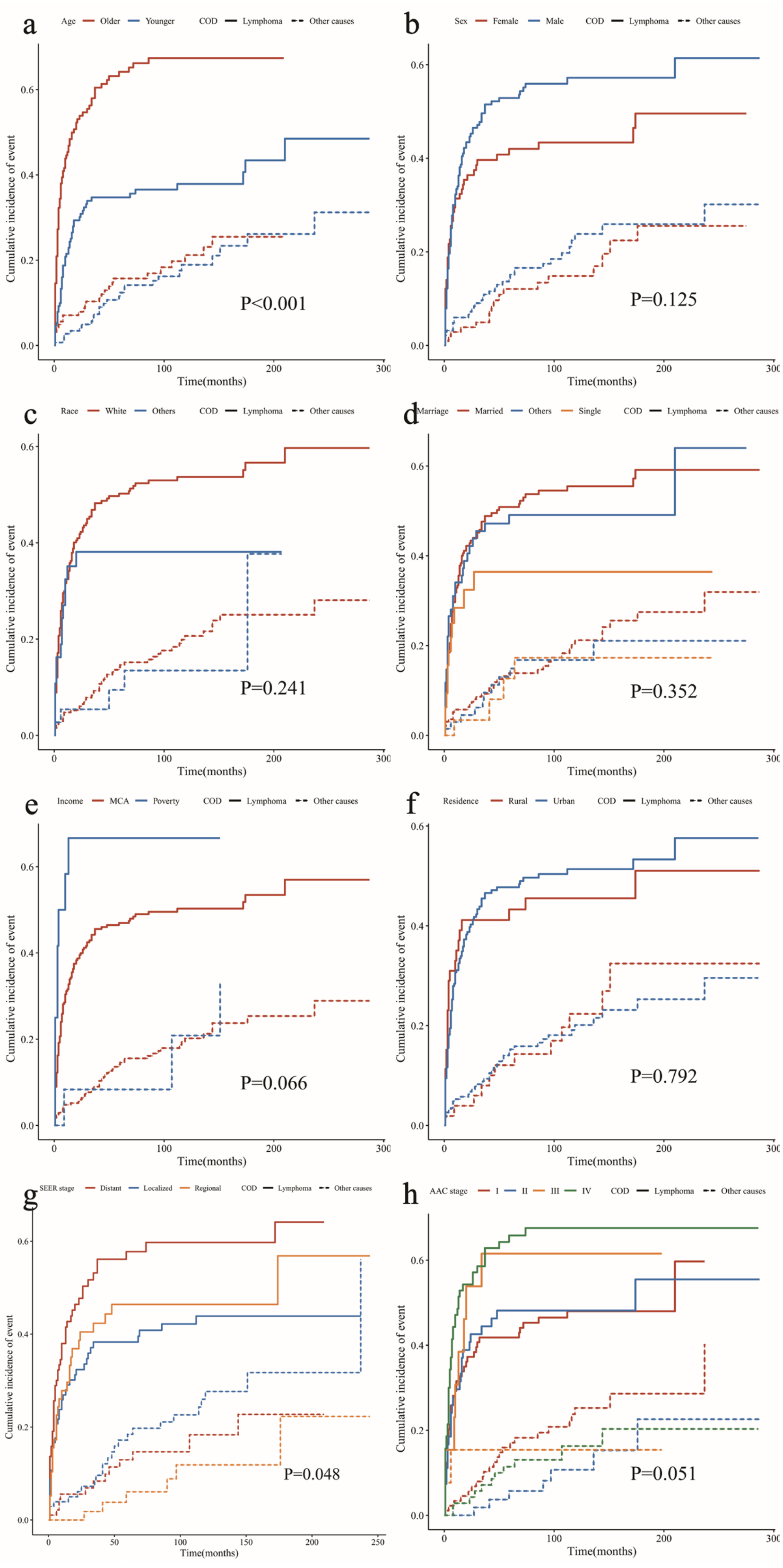

Supplementary Figure S6. Fine-Grey Model for patients based on:
(a) Age;
(b) Sex;
(c) Race;
(d) Marriage;
(e) Income;
(f) Residence;
(g) SEER stage;
(h) AAC stage.

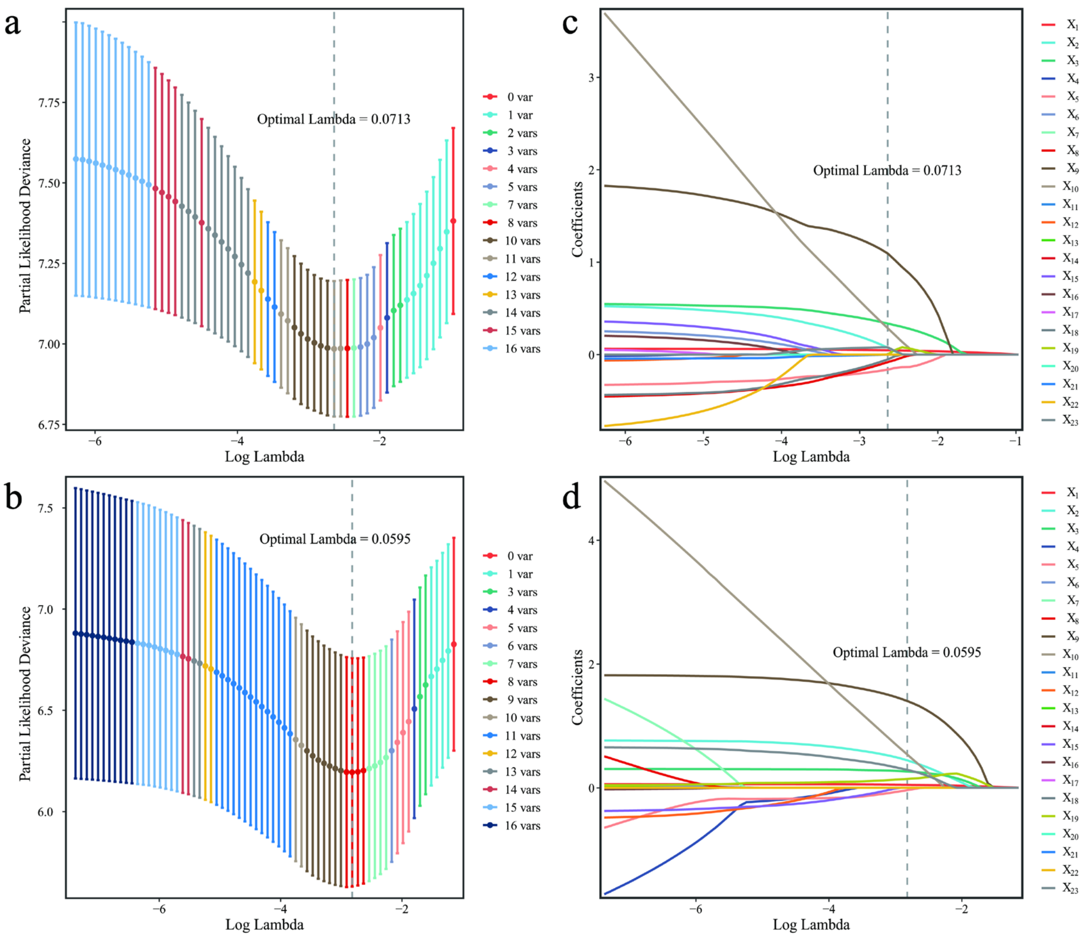

Supplementary Figure S7. LASSO regression.

(a. The 10- fold cross-validation results of OS;
b. The 10- fold cross-validation results of DSS;
c. LASSO coefficient profiles of the 23 variables of OS;
d. LASSO coefficient profiles of the 23 variables of DSS.)
